# A Study on the Mechanical Characteristics and Self-Preservation Performance of a Deployment Mechanism with a Large Exhibition Ratio during Its Gathering Process

**DOI:** 10.3390/ma13071650

**Published:** 2020-04-02

**Authors:** Ning Kong, Jinyu Li, Congfa Zhang, Jie Zhang, Hongbo Li, Haowei Wang, Biao Li, Yuan Wang

**Affiliations:** 1 School of Mechanical Engineering, University of Science and Technology Beijing, Beijing 100083, China; hblglijinyu@163.com (J.L.); zhangjie@ustb.edu.cn (J.Z.); lihongbo@ustb.edu.cn (H.L.); 2 Beijing Institute of Spacecraft System Engineering, Beijing 100094, China; wanghaowei000@126.com (H.W.); cast_libiao@163.com (B.L.); 3 Beijing Institute of Space Launch Technology, Beijing 100076, China; apwangyuan1989@163.com

**Keywords:** deployment mechanism, mechanical characteristics, self-preservation mechanism, stability, gathering process

## Abstract

In order to study the stress state and stability of a spiral tube and actuator for controlled extension and retraction (STACER) during the launching process of a satellite, finite element software was applied to establish a finite element model of STACER via the explicit dynamics analysis method. The influence of top rod’s radius on the gathering or so called packaging process of STACER was analyzed. The effects of surface friction coefficient and acceleration on the stability were studied during the gathering process. It was found that the top rod radius directly affects the gathering load and the deformation around the rivet of the STACER. When the spring reel is gathered, the friction coefficient between contact surfaces, and the acceleration, work on the stability of STACER. The stability of STACER can be maintained by a friction coefficient with small fluctuations. An unstable state occurs after the STACER is gathered when the direction of acceleration is parallel to the axial direction of the rivet. A mechanical test on the STACER is conducted to verify the reliability and accuracy of the model. The force trend is similar between the finite element result and experimental result. This work will contribute to the theoretical development for designing the radius of the top rod of the spring reel, the surface friction coefficient of the STACER and the position of the spring reel during the launch process of satellites.

## 1. Introduction

**Figure 1:**
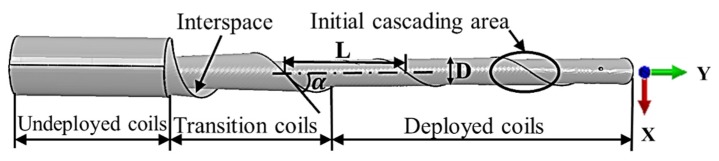
The deploying process of the spring reel of STACER.

In the aerospace sector, there is an increasing demand for a deployment mechanism that can be used in areas such as satellite communications, space science and deep space exploration [1,2,3,4]. The spiral tube and actuator for controlled extension and retraction (STACER) mechanism is widely used in the aerospace industry due to its advantages, such as a large exhibition ratio, strong thermal symmetry along the ring, and a simply equipped structure. Over 650 STACER units have been used in the past 30 years [5,6,7,8]. As shown in Figure 1, it is a layered elastic circular tube structure which is spirally deformed with a thin metal strip at a fixed inclination angle. In the deploying process through its own elastic potential energy, the STACER achieves a rigid support character due to the tight contact of the strip layers [9].

A typical deployment mechanism with a large exhibition ratio includes STACER, a tape spring and the storable tubular extendible member (STEM) [10,11,12,13]. The current research on such a mechanism mainly focuses on the optimization of configuration parameters, the analysis of mechanical characteristics during the deploying and gathering processes, and the influence of environmental factors on its stability [14,15,16,17]. According to the configuration characteristics of the tape spring, three kinds of gathering forms are proposed. Seffen [18] studied the deploying process of one-dimensional and two-dimensional gathering forms and found that the tape spring would form an elastic gathering region with zero transverse curvature and constant longitudinal curvature. According to this morphological feature, the relationship between the bending moment and the rotation angle of the bending region is obtained. Soykasap [19] studied the influences of the shape parameters, such as the curvature, thickness, and wrap angle of the tape spring, on the peak bending moment, steady bending moment, and strain during the bending process in order to optimize these parameters of the tape spring according to the influence tendency. Rimrott and Fritzsche [20] pointed out that the standard ratio of diameter to wall thickness when designing a STEM is limited by the yield strain of the material. The properties of the STEM cross-section (centroid, shear center, moment of inertia, etc.) are affected by the envelope angle of the section. During the configuration of an optimization design, the mechanical properties of different configurations during the deployment process are quite different. The differences may determine whether the deployment mechanism can be deployed successfully. Zhang [21] established a nonlinear explicit dynamics finite element model for studying the process of STEM expansion. The effects of different curling methods and curl tightness on the unfolding process were studied. The positive curl and tight curl were beneficial to the deployment process of STEM. Aridon [22,23] found that the displacement of the tape spring and the thrust curve presents significant hysteresis characteristics after the coil spring is gathered in a winding manner through both experimental and simulation methods. It also determines whether the entire mechanism can be successfully deployed. The impact arm designed by Ullrich [5] based on STACER mechanism proves that the thrust force is constant during the deployment of STACER. It indicates that the STACER mechanism is essentially a constant force spring. It is also one of the criteria for judging whether a STACER mechanism is qualified for employment. Wu [9] proposed to establish a multi-body flexible dynamics simulation analysis model of STACER, and fully studied the deformation process, load state, and energy variation law during its deployment. Through theoretical analysis, a simplified formula for calculating the thrust force was proposed, which provides a theoretical support for the design and application of the deployment mechanism. The deployment of the STACER is influenced by its own configuration design and the deployment environment. Factors of the environment of space include its being a vacuum, microgravity, extreme cold and high temperatures, etc. Extreme environmental conditions tend to reduce the stability of the space-bound mechanism [24,25,26,27]. In the study of the impact arm, Bougeret [28] designed a prototype flight experiment and established a vertical vacuum laboratory. It is applied to verify whether the vibration frequency and deployment force of the impact arm meet the requirements during the deployment process in the space environment. This test well considers the influence of microgravity and vacuum environment on STACER during its deployment process. The test conditions are close to the space environment, and the reliability is high, but the cost is huge.

The research of above-mentioned with thin-walled expansion mechanism mainly focuses on the mechanical properties analysis of the deployment process. For STACER, which requires repeatable deployment, the stability analyses are equally important during the gathering process and after gathering. STACER needs to complete the process of packaging and deploying alternately due to different working conditions [29,30]. When the packaging process occurs, different layers are misaligned. If the outer layer finally gathers higher than the inner layer, while the size of the outer layer exceeds the storage bin, the subsequent work of the space equipment may not be started or the STACER cannot be expanded again. It will directly lead to the failure of the task. Self-preservation is defined as the state of the STACER structure being completely stored in the storage bucket and not tending to deploy with a stable state. Therefore, it is necessary to conduct research on the self-preservation of STACER during its gathering process and after gathering. The thin-walled deployable mechanism shows the characteristics of large deformation, large displacement, and large rotation, and there is a large amount of overlapping contact and friction between the layers, which is difficult for the dynamic simulation [31]. Based on the work mentioned above with thin-walled deployable mechanism, the finite element model of nonlinear explicit dynamics of STACER mechanism is established by finite element software Abaqus. The mechanical properties and self-preservation mechanism of the STACER are studied during its deploying process. It will provide both theoretical and application guidance for its further development.

## 2. STACER Theoretical Model

**Figure 2:**
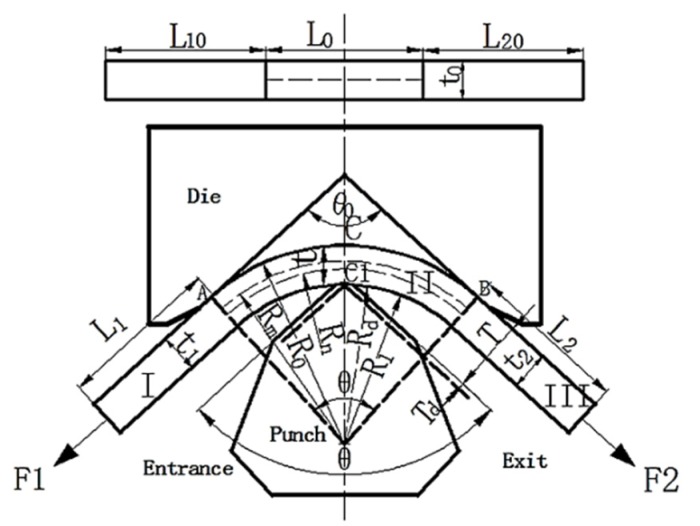
Geometric parameters and area division of strip CSPB forming process.

Consider in this study in the scope of materials and its processing technology, an analytic model of the forming process of the STACER cylinder is proposed. It aims to provide a general method for the design and production of a STACER from a theoretical perspective. The key size parameters of STACER include gradient diameter D, helix angle α, and pitch L, as shown in Figure 1 [32]. In Figure 2, the helix angle α is determined by the angle at which the strip is placed between the die. The gradual diameter D and the pitch L are influenced by various factors, including material and forming process parameters.

STACER is produced by a compositing stretch and press bending (CSPB) process. The forming principle is shown in Figure 2. The strip enters from the left side at an angle α to the mold, is plastically deformed by the mold, and is collected on the right side. This process can be divided into three areas: I is an elastic deformation zone, which is subjected to tension F1 and remains straightened without deformation. Zone II is a plastic deformation zone, which the strip is bent and deformed by the action of convex and concave molds and tension on both sides. Zone III is a complex deformation area; the strip is straightened backwards in the exit’s direction [33,34].

In order to establish a theoretical model for STACER, the theoretical calculation hypothesis is proposed:

1. The strip used for CSPB forming is a thin strip with a thickness direction much smaller than the width direction. It is considered that the strain in the width direction during the bending process is 0.
2. The tangential tensile stress on the strip width direction is evenly distributed along the thickness direction. The tensile stress exists to reduce the thickness of the strip and lead to the curved neutral layer moving inward.
3. The strip is subjected to the Kirchhoff straight normal assumption during the bending forming process. The Bausching effect is neglected during the strip forming process.
4. The strip has a constant volume during the CSPB forming process [35,36,37].

Based on the above assumptions, a mathematical model of STACER is established. During the forming process of the material by the CSPB method, the effect of the convex and concave mold on the sheet material can be simplified as the effect on the three points A, B, and C on the strip. Considering the punch radius $R_{d}$, the action point on the sheet is moved downwards and the radius of curvature has been increased. The change in mold fillet can be completely equivalent to the change of mold gap. The equivalent mold clearance is:

(1) $$T_{e}=T+T_{d}=R_{d}(1-\sin\frac{\theta_{0}}{2})+T,$$

Among them, T and $T_{d}$ are the incremental gaps between the mold gap and the mold radius.

The bending angle θ is related to the mold cone angle $\theta_{0}$ as follows:

(2) $$\theta =\pi -\theta_{0},$$

From the formula above, the increase of the mold angle increases the gap of the equivalent mold, and the diameter of the obtained spring reel is increased.

Assuming that the strip has a thickness t after forming, the relationship between the outer radius of curvature $R_{0}$ and the inner radius of curvature $R_{1}$ of the strip after forming is as follows:

(3) $$R_{0}=R_{1}+t=R_{1}+t_{0}-\Delta t,$$

(4) $$R_{m}=\frac{1}{2}(R_{1}+R_{0}),$$

where $t_{0}$ is the initial thickness of the strip and Δt is the thickness increment before and after the strip is deformed. $\Delta t=t_{0}-t$. According to the geometric relationship between parameters, it can be found that:

(5) $$R_{0}=T_{e}+R_{1}\sin\frac{\theta_{0}}{2},$$

From Equations (2)–(4), it can be calculated:

(6) $$R_{1}+t=T+R_{d}(1-\sin\frac{\theta_{0}}{2})+R_{1}\sin\frac{\theta_{0}}{2},$$

Solving is available:

(7) $$R_{1}=\frac{T-t}{1-\sin\frac{\theta_{0}}{2}}+R_{d},$$

Bringing Equation (7) into Equation (3), we can obtain:

(8) $$R_{0}=\frac{T-\mathrm{tsin}\frac{\theta_{0}}{2}}{1-\sin\frac{\theta_{0}}{2}}+R_{d},$$

The mathematical relationship between the curvature radius of the inner and outer layers of the strip with other factors is not able to be considered through Equations (6) and (7). The strip reduction during rolling is not considered. When considering strip reduction, Δt≠0, the following relationship can be obtained from the assumption that the volume is constant before and after deformation of the material:

(9) $$\left\{ \begin{array}{l} L_{10}t_{0}=L_{1}t_{1}\\ L_{0}t_{0}=\frac{\theta }{2}(R_{0}^{2}-R_{1}^{2})=R_{m}\theta t\\ L_{20}t_{0}=L_{2}t_{2} \end{array}\right.,$$

In the equation, $L_{10}$, $L_{0}$, and $L_{20}$ are the original lengths of the three regions before processing; $L_{1}$ and $L_{3}$ are the strip lengths corresponding to Zones I and III after processing; $t_{0}$ is the initial thickness of the strip; $R_{0}$ and $R_{1}$ is the inner and outer radii of the strip, respectively; $R_{m}$ is the curvature radius of the mid-section of the strip; θ is the angle of curvature; $R_{n}$ is the curvature radius of the neutral layer. The following relationship can be obtained:

(10) $$R_{m}=\frac{1}{2}(R_{0}+R_{1}),$$

(11) $$R_{n}=\frac{L_{0}}{\theta },$$

According to the R. Hill principle, for an ideal strip, when elastic and plastic deformation occurs, the bend radius of neutral layer of a strip is:

(12) $$R_{n}=\sqrt{R_{0}R_{1}},$$

In summary, after the simultaneous calculation of Equation (7), Equation (9), Equation (10), and Equation (11), Equation (13) can be obtained as follows:

(13) $${\left(\frac{T-t}{1-\sin\frac{\theta_{0}}{2}}+R_{d}\right)}^{2}+\left(\frac{T-t}{1-\sin\frac{\theta_{0}}{2}}+R_{d}\right)t=\frac{t^{4}}{4\left(t_{0}^{2}{-t}^{2}\right)},$$

The geometric relationship between the strip thickness t after passing through the CSPB process and the convex fillet $R_{d}$, the gap T, the curvature R of the STACER, and the taper angle $\theta_{0}$ of the mold can be obtained in consideration of the strip reduction.

The key geometric dimensions of the STACER include the helix angle α, the pitch L, and the diameter D. In the theoretical case, according to the equations above, STACERs with different sizes can be designed. The STACER discussed in this paper is a STACER prepared with a post-tension of 10 N, a punch radius of 0.5 mm, a strip thickness of 0.15 mm, a bandwidth of 20 mm, and an angle between the strip and the mold of 60°. α is 60°, L is 127 mm, and D is 18.6 mm at the smallest diameter.

## 3. Finite Element Model of STACER

### 3.1. Design of the STACER Model

The STACER is a constant force spring reel, which is geometrically understood to be formed by a thin wall strip rotating at a certain pitch and helix angle, as shown in Figure 1. Each of STACER layers presents characteristics with equal natural curvature. The diameters are equal everywhere without external constraints and self-constraints. The STACER shows a constant force property that maintains a constant mechanical output during deployment and gathering processes. The thin walls are spirally covered layer by layer and increase the diameter. It shows an equidistant spiral when projected from the small diameter end to the large end. According to this geometrical feature, a modeling method is proposed for STACER.

**Figure 3:**
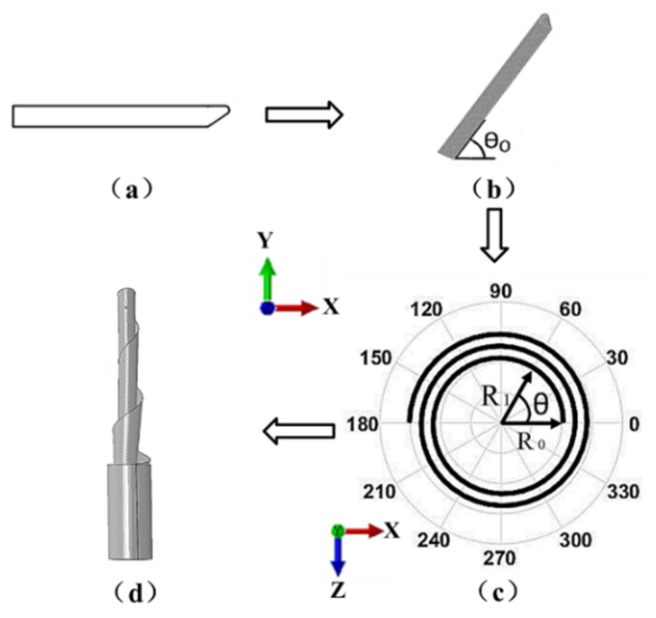
Geometric model of STACER (**a**) strip; (**b**) coordinates; (**c**) top view after curling and (**d**) spring reel.

The strip model in Figure 3a is rotated around the Z axis by $\theta_{0}$ and meshed, as shown in Figure 3b. Then, the strip is curled over and the top view after curling (projection of the helix in the xz plane), as shown in Figure 3c. The relationship between the helix radius and the rotation angle is shown in Equation (14).

(14) $$\begin{array}{l} L=\int_{0}^{\theta }\left(R_{0}+t\frac{\theta }{2\pi }\right)d\theta \\ R_{1}=R_{0}+t\frac{\theta }{2\pi } \end{array},$$

In the equation, L is the arc length; $R_{0}$ is the minimum radius position; t is the strip thickness; θ is the rotation angle; $R_{1}$ is the radius of corresponding position with θ.

In Figure 3b, the coordinates of each node on a flat strip after meshing can be transformed by Equation (14) to obtain new coordinates for the spring reel, as shown in Figure 3d.

In order to change the cumbersome problems of repeated modeling for the STACERs with different sizes, a parametric modeling method is proposed for STACERs with different diameters of the same series. The strip node of STACER can be directly replaced by the model description file.

### 3.2. Finite Element Model of STACER

**Figure 4:**
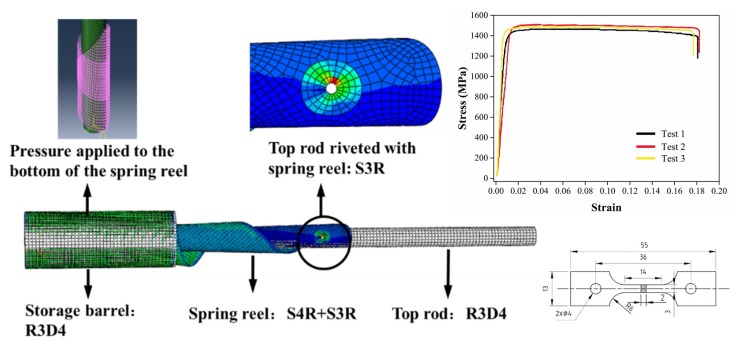
Finite element model of STACER with stress–strain curves and specimen geometry.

The STACER consists of three parts, the top rod, the spring reel, and the storage barrel, as shown in Figure 4. The spring reel and the top rod are riveted. The top rod and the storage barrel are rigid bodies, and the R3D4 rigid body element is selected without deformation. The model is applied with a displacement on the top rod, which only has a freedom to move downwards. The purpose of the displacement is to push the spring reel to be gathered. The top rod pulls the spring reel and controls its extension length. The size is 300 mm in length with a radius of 9.3 mm. The storage barrel is a storage device for STACER with a length of 130 mm and a radius of 25 mm.

The structural parameters of STACER are shown in Table 1. Since its wall thickness is only 0.15 mm with a width of 127 mm, it is much smaller than the width dimension (generally less than 1/10). The thickness direction stress can be neglected and the shell element is selected to simulate this structure. In the modelling process, the spring reels are divided with three mesh sizes of 0.99075, 2.06864, and 3.03555 mm. These three sizes of mesh are applied for the mesh sensitivity study, as shown in Figure S1. The simulation results are shown in Figure S2. The variation trend of the force exerted on the top rod is similar for all three cases. It proves the model is not quite sensitive to the mesh size and presents high reliability for the simulation with various mesh sizes. Therefore, a mesh size of 2.06864 is selected with consideration of both the calculation accuracy and computational efficiency. In this work, a larger mesh is selected for the less concerned area, and the meshes which are near the area between the spring reel and the top rod are refined in order to achieve both efficiency and precision for the model. When selecting the element type, the meshes of the spring reel and the top rod connected with the concentrated force are refined, and the S3R (3-node triangular general-purpose shell) element is selected. The remaining part is set as a S4R (4-node thin shell) element. The total number of elements in this model is 52,349, which includes 13,419 R3D4 (3-node 3D rigid triangular facet) elements and 38,930 S3R and S4R elements. The development of STECER is based on its special materials property and materials processing technology. The material used for the STACER is Co_40_CrNiMo alloy. The Co_40_CrNiMo alloy is a kind of high elastic alloy, which has been widely used in elastic components because of its high strength, high elastic limit, low elastic aftereffect, and acid and alkali corrosion resistance. The Co_40_CrNiMo alloy strips used in this study ere produced by hot rolling, annealing, and cold rolling. The structure of Co_40_CrNiMo alloy strip is FCC at room temperature. Due to the existence of Co element, the stacking fault energy of the alloy is relatively low, and a large number of extended dislocations and twins are easily produced during deformation. The extended dislocation is composed of two Shockley dislocations and a layer of stacking fault sandwiched in the middle. The stacking fault zone of the FCC Co_40_CrNiMo alloy is the HCP structure [38]. Some of the annealing twins are staggered, and the network structure formed by the fine deformation twins hinder the dislocation movement, which can significantly improve the strength and provide conditions for the aging strengthening of the alloy. Cold deformation makes the strength of the alloy increase significantly, and twinning is easy to occur during cold rolling [39]. At the same time, high dislocation density can strengthen the alloy. All these microstructures contribute is responsible for the production of a required STACER. The tensile specimen geometry and stress–strain curves of the Co_40_CrNiMo alloy are shown in Figure 4. The tensile tests were carried out on a DDL50 testing machine (Changchun mechanical research institute, Changchun, China) in the longitudinal direction. The tensile tests were repeated three times to obtain the stress–strain curves. The yield strength, tensile strength, and elastic modulus were 1291 MPa, 1452 MPa and 205 GPa respectively. The deformation of the STACER was conducted by a method of continuous bending forming under tension and compression conditions. Based on the press bending mold, the tension is applied on both sides of the strip, while the strip is pulled out from one side. When the strip is inserted at a certain angle at the normal orientation to the mold, the forming of the STACER can be achieved from a strip. It is quite important that all the functions and actions of the STACER closely rely on the material’s properties and deformation method.

This model needs to ensure that the top end of the spring reel is connected to the top rod, and the bottom end of the reel is in contact with the storage barrel. The spring reel and the top rod connection position is required to move together and satisfy the mutual rotation relationship. It is necessary to establish a local coordinate system for the riveting joint and the storage barrel respectively, so that the outward movement in the diameter direction can be achieved at the contact position of the bottom end of the reel and the storage barrel. There is a small hole at the top of the spring reel, and the center of the hole is the riveting position of the spring reel and the top rod. A reference point is established at the center of the circle in order to ensure that the center of the circle can be rotated. The reference point and each point round the hole are coupled and constrained. It is necessary to perform a coupling constraint on the riveting position with a local coordinate system in order to ensure that the constrained point can be rotated.

The gathering process of STACER is a typical nonlinear process with a large deformation and complicated calculations. Therefore, the explicit computing method is used and the mass amplification factor is set to increase the calculation speed. The model establishment process is appropriately simplified, considering only the contact friction between the inner and outer layers of the spring reel. The simplification on the STACER model includes the contact state of the outermost spring reel and the wall of the storage barrel. In the process of simplification, the contact state of the outermost spring reel and the wall of the storage barrel is simulated by applying the pressure between the spring reel and the wall of the storage barrel. A rigid and elastic model is applied to study the elastic deformation of the STACER. That is due to the requirement that the mechanism is used in its elastic stage. When the stress exceeds the yield stress of Co_40_CrNiMo alloy in the simulation, the plastic deformation may occur on the spring reel of a STACER. It leads to a failure on the packaging or deploying process so as to lose its function. When the model is calculated, it will be affected by a convergence, which is not beneficial for the results. As long as the step size is small enough, the calculation can be achieved. However, the explicit algorithm takes a longer time for the simulation.

The actual spring reel is in a state of layered phase with pressure. The model of the STACER is simplified compared to the actual model. In order to reduce the influence of the state of outermost reel layer on the inner reel, pressure is applied to the outermost reel portion of the STACER in order to ensure it completely contacts to the wall of the storage barrel, as shown in Figure 4. A gathered model is established to analyze the factors that affecting the gathering process of the STACER.

The validation of the numerical model correctness was conducted by both the simulation phenomenon on the state of the STACER and the tests. For the simulation validation, it is focused on the storage characteristics in the early stage of modelling process. Whether the top of STACER can be moved down smoothly and whether the bottom of STACER can enter the storage bucket are standards for the correct establishment of the model. It is a simulation result validation on a physical structure configuration of the STACER. In the tests, the force which has been registered for a top rod can be compared with the simulation results on both the value and tendency. The force on the top bar can represent the force to the spring reel during the gathering process. In the simulation process, the top rod is defined as a rigid body and a reference point for the modelling establishment, for which it is easy to achieve a data output. It also indicates the materials properties of the STACER under load. Moreover, in the test process, the force on the top rod is easier to measure. Therefore, the force analysis on the top rod is selected for both the simulation and test.

## 4. Analysis of the Factors Affecting on the Gathering Process and Self-Preservation of the STACER

For the FE modelling, the effects of friction coefficient, radius of the top rod, and acceleration on the mechanical characteristics of STACERs have been studied during its packaging process. A plane test method was carried out for the friction coefficient measurement. It was to contact two measured surfaces in a horizontal plane. A certain pressure was applied and the surface was dragged with a constant speed in the horizontal direction. Then the friction coefficient was obtained from a calculation. From the measurements, the friction coefficient ranged from 0.08–0.12. Considering the influences of the environment, such as high temperature or low temperature, in space the friction coefficient is selected as 0.1. Due to the layout requirement, only a maximum radius of 9.3 mm is allowed for the top rod application in a STACER. The only physical hardware of top rod is at a radius of 9.3 mm currently. In the simulation, the extended radius range was selected from 8.7 to 9.3 mm for the top rod investigation. Other specifications still need to be developed in further studies. This study provides a guidance for the effect of top rod radius on its application performance. It also can be used to evaluate the design rationality for the top rod with different radius. The acceleration data of 100 G with different directions in this work is also based on a comprehensive consideration of the rocket’s ability, vibrations, and the severe dynamic impact on the STACER with a large safety factor during the launching process from engineering experience. During the gathering process, the spring reel is drawn downwards by the top rod, so that the reel can be completely stored. According to the design requirements, there are three options for the radius size of top rod. It is necessary to study the influence of the top rods size on the gathering process of the spring reel so as to determine the optimum size. In order to investigate the factors affecting on the self-preservation of a gathered STACER, the acceleration and friction coefficient between layers of the spring reel are studied to explore the stress distribution and topography of STACER under different boundary conditions.

### 4.1. Influence of the Top Rod Radius on the Gathering Process of the STACER

**Figure 5:**
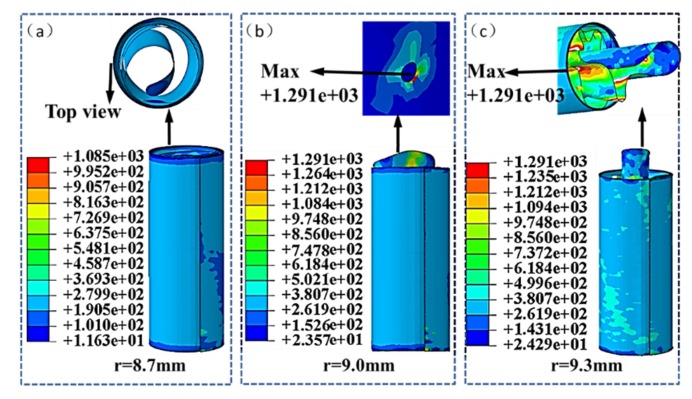
The stress distribution of spring reels during gathering process with top rod radius of (**a**) 8.7 mm, (**b**) 9.0 mm and (**c**) 9.3 mm.

The STACER can be applied as a part of satellite antenna. Due to the installation requirements, the top rod radius can be selected from 8.7, 9.0, and 9.3 mm. The spring reel and storage barrel remains at a certain size. The storage barrel is set with a radius of 25 mm, a height of 130 mm, and a wall thickness of 1 mm. Three kinds of finite element models of STACERs of the mentioned top rod size are built to study the radius’s effect on the gathering process of the STACER. The situation results can be seen in Figure 5a–c. The stress type is von Mises and the unit is MPa.

In all three cases, the full gathering state can be achieved only when the top rod radius is 8.7 mm. The maximum stress state is 1085 MPa, which is less than the material yield strength of 1291 MPa. It shows that the STACER does not undergo plastic deformation during the process of gathering, which satisfies the working requirements for the spring reel. When the radius of the top rod is changed to 9.0 mm, the maximum stress is more than 1291 MPa, which reaches the yield strength of the spring material. It can be seen from the figure that partial plastic deformation occurs in the surrounding portion of the rivet joint. When the radius of the top rod is 9.3 mm, STACER undergoes even higher stress and extensive plastic deformation. The STACER tends to be damaged.

**Figure 6:**
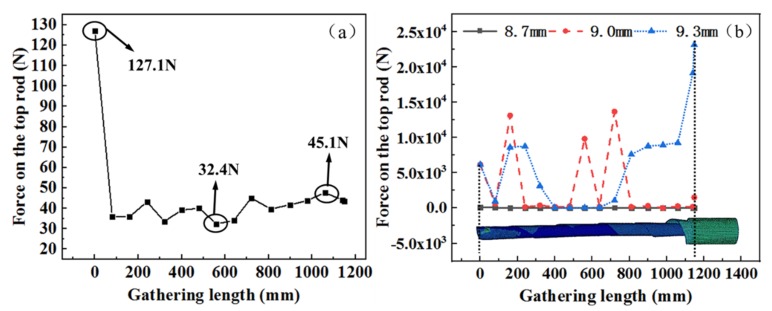
Force diagram during the gathering process on the top rod with (**a**) a radius of 8.7 mm; (**b**) different radial dimensions.

In order to study the force evolution tendency of the top rod during gathering process, the relation between the gathering length and the force on the top rod is analyzed, as shown in Figure 6a,b. STACER is a spring which requires constant force when gathering and deploying. Along with the gathering position, the force analysis at the rod top is carried out for the STACER. When the top rod radius is 8.7 mm, the force on the top rod is generally stable, and the force fluctuates between 32.4 and 45.1 N (do not include the initial loading stage). As the top rod moves downwards, the friction is weakened between the layers of the spring reel. The friction force is reduced while the elastic restoring force gently increases. When the top rod radii are 9.0 and 9.3 mm, the force fluctuates frequently and greatly. The force on the top rod can not be kept constant for the STACER. This is the reason that some STACERs are plastically deformed, as shown in Figure 5.

**Figure 7:**
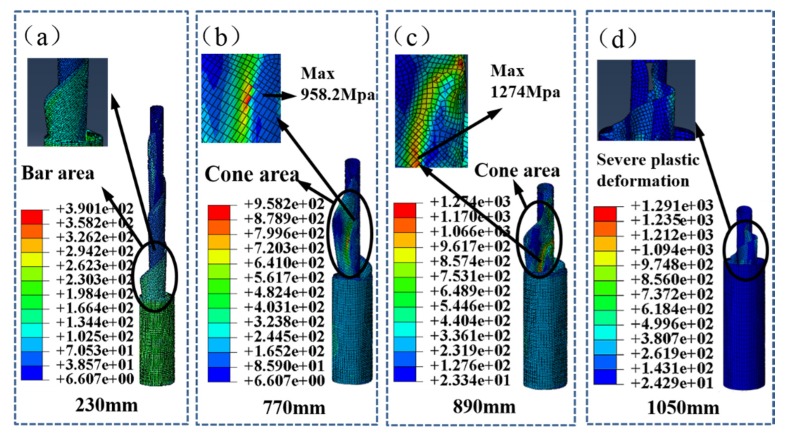
Gathering process with the top rod radius of 9.3 mm when the gathering length is (**a**) 230 mm; (**b**) 770 mm; (**c**) 890 mm; or (**d**) 1050 mm.

The mechanical model for analyzing the gathering process of the top rod with a radius of 9.3 mm is shown in Figure 7a–d. When the gathering length is from 0 mm to 770 mm, the STACER is gathered smoothly and the cone angle is small, as shown in Figure 7a. The transition section presents a cylindrical shape. At 770 mm, the transition section produces a large tapered area, and stress concentration occurs at the tapered area without local shell buckling. With the increase of gathering length at 890 mm, local shell buckling occurs, as shown in Figure 7c. It leads to a failure of the material and even destruction in the subsequent gathering process. At the same diameter of storage barrel, the larger the top rod diameter, the larger the conical degree for the spring reel when the gathering distance is the same. This taper greatly increases the friction between the layers of the spring reel so as to block the gathering process. Therefore, the top rod radius is chosen as 8.7 mm in order to avoid possible deformation.

### 4.2. Effect of Layered Friction Coefficient on the Self-Preservation Performance of STACER

**Figure 8:**
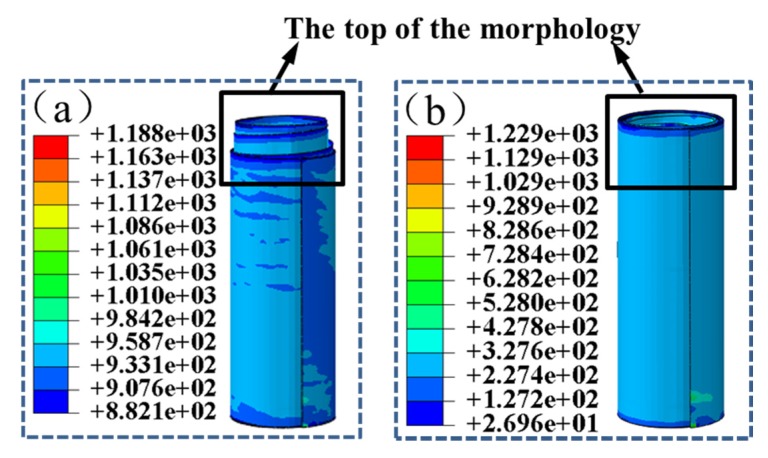
The effect of friction coefficients on the top morphology of the spring reel when (**a**) µ = 0.009; (**b**) µ = 0.025, 0.05, 0.1, and 0.3.

From the analysis above, the radius of the elastic spring reel is selected to be 8.7 mm. During the launch process of satellites, the spring reel may be affected by the extreme conditions, such as high temperature and microgravity, which may change the friction conditions between the layers of the spring reel. Therefore, it is necessary to investigate the effects of friction coefficient on the self-preservation of the STACER. From the experiment [28], the friction coefficient is around 0.1 at room temperature on the earth. The friction coefficient was also determined by the experimental validation. The friction coefficient was about 0.1 in the test. For the simulation, the friction coefficients was set to 0.009, 0.025, 0.05, 0.1, and 0.3 in case a sudden change happens on the friction coefficient of a STACER. Taking the gathered state of the spring reel as the initial state, the effect of friction coefficient between the layers of the spring reel on the self-preservation performance of the reel was studied. The simulation results are shown in Figure 8. The spring reel shows an unstable state only when the friction coefficient is 0.009, as shown in Figure 8a. The STACER is completely gathered with a stable state and the maximum stress value is still under the required stress condition under all other friction coefficient conditions, as shown in Figure 8b.

**Figure 9:**
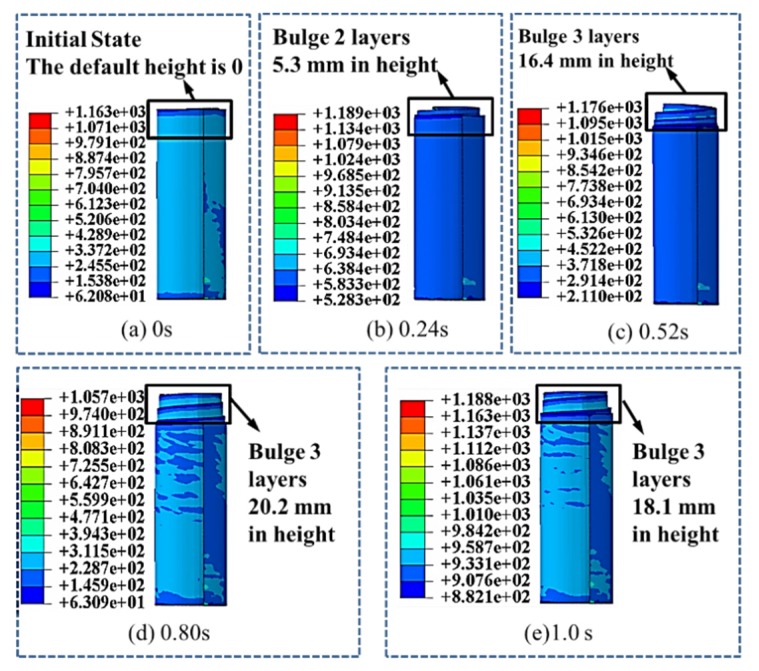
Evolution of top morphology of spring reel when the friction coefficient is 0.009 at (**a**) 0 s; (**b**) 0.24 s; (**c**) 0.52 s; (**d**) 0.80 s and (**e**) 1.0 s.

When the friction coefficient is 0.009, the evolution of top morphology is shown in Figure 9a–e. At the beginning, the spring reel is completely gathered and the bulge height of the spring reel is 0. From 0.24 s, two layers begin to exceed the top of the storage barrel and keep rises until the maximum height is reached at 0.80 s with a height of 20.2 mm. Then the bulge height is slightly decreased and when at 1.0 s, three layers exceed the barrel top and the bulge height is 18.1 mm.

**Figure 10:**
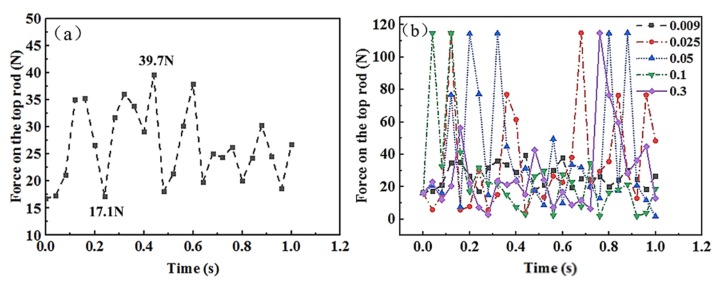
Force diagram of top rod with (**a**) a friction coefficient of 0.009 and (**b**) different friction coefficients.

The effect of friction coefficients on the force at the top rod is further analyzed, as shown in Figure 10a. The force on the top rod fluctuated between 17.1348 and 39.6567 N with a small changes when the friction coefficient was 0.009. As shown in Figure 10b, the force on the top rod reaches a peak with the maximum value around 115 N when the friction coefficient increases from 0.009. The reason is that when the friction coefficient changes, the spring reel reaches its balance state, and the relative movement between the layers occurs. Then the friction force is generated which leads to a force fluctuation at the top rod. It can be concluded that the changes in friction coefficient show limited effect on the stability and self-preservation performance when the friction coefficient is between 0.009 and 0.3.

However, when the friction coefficient is equal to or less than 0.009, the friction force between the spring reel layers cannot resist the elastic force from the spring reel. Some layers move and exceed the top of storage barrel. The initial state of the spring reel with a stable state is destroyed. Therefore, consideration should be taken when a spring reel is applied with a friction coefficient below 0.009 between layers. Therefore, when the STACER works, the spring reel deploys from the storage barrel, and the friction coefficient will affect the deploying process. The optimum friction coefficient range should be designed in consideration of the spring reel shape and its stress distribution.

### 4.3. Influence of Acceleration on the Gathered State of STACER during Launching Process

In an actual launch process, the STACER is fully packaged and fixed in place so as to avoid severe plastic deformation. However, it still undertakes a large acceleration in the launching process with an unstable state between spring layers. A problem occurred on the STACER during a successful deployment. A jamming of the mechanism occurred after the ground tests with extra accelerations. This is the reason for the acceleration study. In the simulation, the spring reel is fixed by both the top rod and storage bucket.

**Figure 11:**
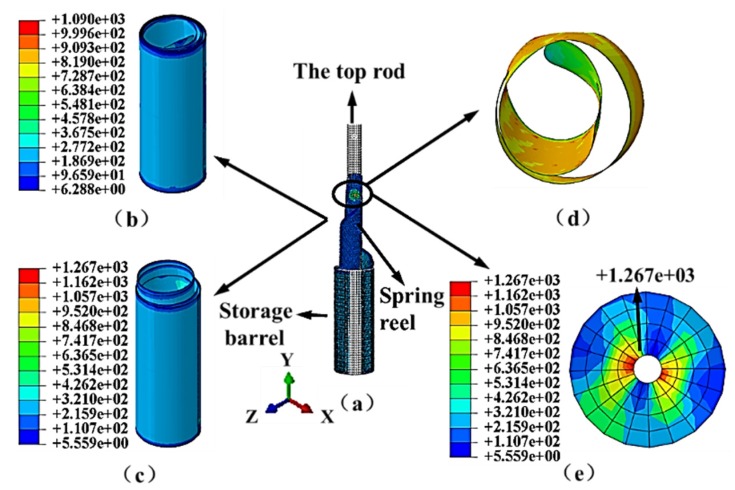
Influence of acceleration on the morphology and stress distribution of the spring reel with a friction coefficient of 0.1 between layers: (**a**) STACER model; (**b**) complete gathered state; (**c**) partial gathered state; (**d**) the morphology of the innermost circle; and (**e**) stress distribution.

The STACER experiences weight loss and overweight during the launch process. The contact conditions change over between the layers of the spring reel, which affects the stability and self-preservation performance of the STACER. Therefore, the purpose for analysis of self-preservation under launch accelerations is to provide a guidance for the installation of the STACER with a safe orientation. It is necessary to explore the effect of acceleration on the stability of the spring reel. From the initial state of the spring reel in a well gathered state, accelerations with different directions are applied to the spring reel. The acceleration of 100 G is selected according to various ground test results and is quite credible for the simulation. Accelerations in six different directions are applied to the spring reel, which are the positive and negative directions of the X-axis, Y-axis, and Z-axis, respectively. Figure 11a–d shows the simulation results corresponding to the accelerations at six directions.

In the analysis of the six boundary conditions, the results are mainly divided into two categories, as shown in Figure 11b,c. When the acceleration directions are along the negative X-axis, positive and negative Y-axis, and Z-axis with 100 G, the spring reel stays stable, as shown in Figure 11b. When the acceleration direction is along the positive X-axis, which is parallel to the axis of rivet with a pressure, the spring reel cannot be completely gathered, as shown in Figure 11c. It can be concluded that the gaps between the layers of the spring reel lead to the partial gathered state of the STACER. When the acceleration is applied along the +X-axis, the morphology of innermost circle is observed, as shown in Figure 11d. The position of stress concentration occurs at the riveting point of the STACER, as shown in Figure 11e. The maximum stress is 1267 MPa, which is less than the yield strength of the spring reel material.

**Figure 12:**
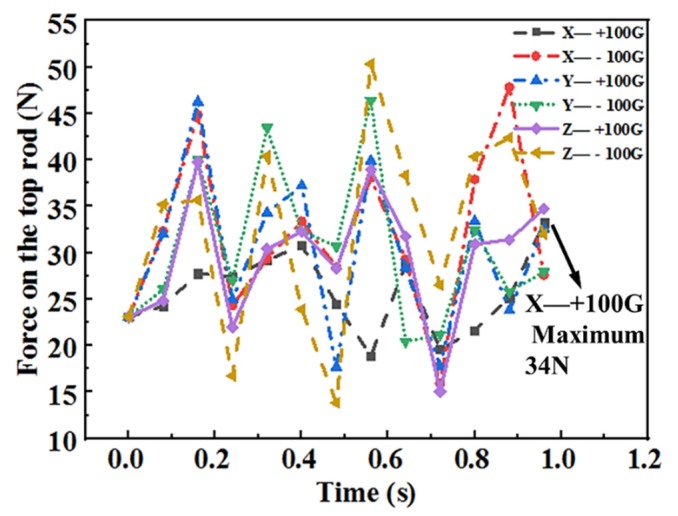
Force diagram of the top rod under different acceleration conditions.

As can be seen of the black line in Figure 12, there was a the force evolution with the acceleration of 100G applied along the positive direction of the X-axis. It is noteworthy that the fluctuation is the actual calculation result on the force evolution of the top rod with different friction coefficient and acceleration. It reflects the whole process of the force changes from the beginning to the end of the gathering process. In this case, the force of the top rod is significantly different from the other five acceleration conditions. The maximum force of the top rod is 34 N. Instead, the top rod is subjected to the trend with multiple peaks and valleys alternately under other five acceleration conditions. The maximum force is between 40 and 51 N, which is much higher than the maximum force with the acceleration along the direction of +X-axis.

**Figure 13:**
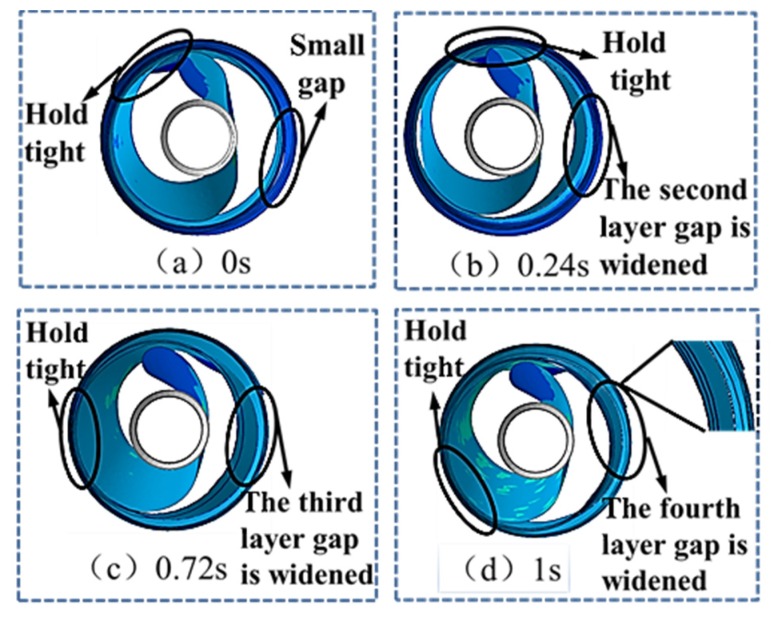
The morphology of the spring reel under the acceleration condition with X-+100G at (**a**) 0 s; (**b**) 0.24 s; (**c**) 0.72 s; and (**d**) 1 s.

When the acceleration is along the positive direction of the X-axis, relative movement occurs between the layers of the spring reel, as shown in Figure 13a–d. The innermost layer near the top rod spreads outward and the gap between the layers gradually increases. The friction force decreases and some of the layers exceed the innermost layer, and then it deploys from the storage barrel. In the other five acceleration conditions, during the movement between the inner layer and the layer of STACER, only limited local areas show intermittent gaps. The friction force of the whole spring reel is stable. The top rod is able to provide sufficient resistance force to avoid the large displacement of the top rod, which keeps the self-preservation and stability of the STACER.

It is concluded that during the satellite launching process, the spring reel should be placed along the direction of the +X-axis so as to reduce the large gap between the STACER layers and maintain good self-preservation performance. Since the friction force is a favorable factor for blocking the relative movement between the layers of the spring reel during the launching process, the friction coefficient is suggested to be increased to a certain extent when the STACER is gathered.

## 5. Test Design and Verification

The FEM simulation is able to provide guidance for the design of a STACER with the consideration of the friction coefficient, radius of the top rod, and acceleration with an optimized selection. In an experiment, the experimental results can be used to verify the accuracy of the model. After that, the verified model can be used to prove other parameters in the test, such as the friction coefficient. With the development of a STACER in serialization, the simulation model can be reused for a further studies with different parameters. After the simulation analysis of the gathering process of STACER was carried out with the top rod radii of 8.7, 9.0, and 9.3 mm, the force evolution of the top rod during the whole gathering process was shown to be small when the top rod radius was 8.7 mm. During the gathering process, the force value is between 32.4 and 45.1 N, which meets the constant force requirement. In order to verify the reliability and accuracy of the model, a mechanical test on the STACER is conducted to study the gathering process of the spring reel with a top rod radius of 8.7 mm.

**Figure 14:**
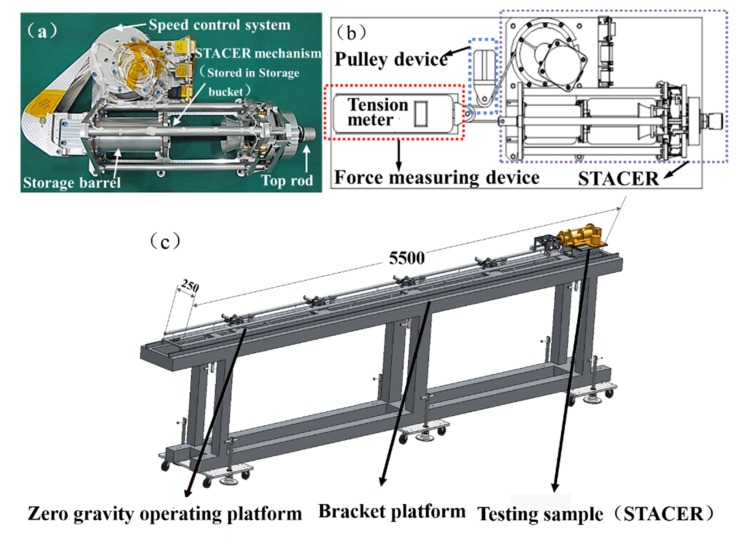
Equipment illustration of (**a**) STACER; (**b**) test diagram; and (**c**) test basement.

Figure 14 is an illustration of the test equipment. The STACER consists of a storage bucket, a spring reel, a top rod, and a speed control mechanism. The top rod radius of the STACER is 8.7 mm. Figure 14a is an actual photograph of the STACER. In this experiment, the top rod force is tested during the gathering process. The test diagram is shown in Figure 14b. The force sensor of the test system is mounted to the rear of the spring reel by a bracket. The top rod is connected to the speed control system by the cable. The force sensor of the test system is installed on the cable by means of stand base. During the gathering process, the force of the top rod is transmitted to the force sensor through the pulley and the cable so as to collect the force data. The actual test basement is shown in Figure 14c. The test basement consists of a zero gravity operating platform, bracket platform, and testing sample (STACER).

The STACER is placed on the zero gravity operating platform. A constant velocity motion is required during the gathering process. The SUNDOO SH-2K sensor is applied for the force testing. The measurement accuracy of the sensor is 0.1 N and the sampling frequency is more than 10 Hz. The system software collects sensor data and generates files automatically.

**Figure 15:**
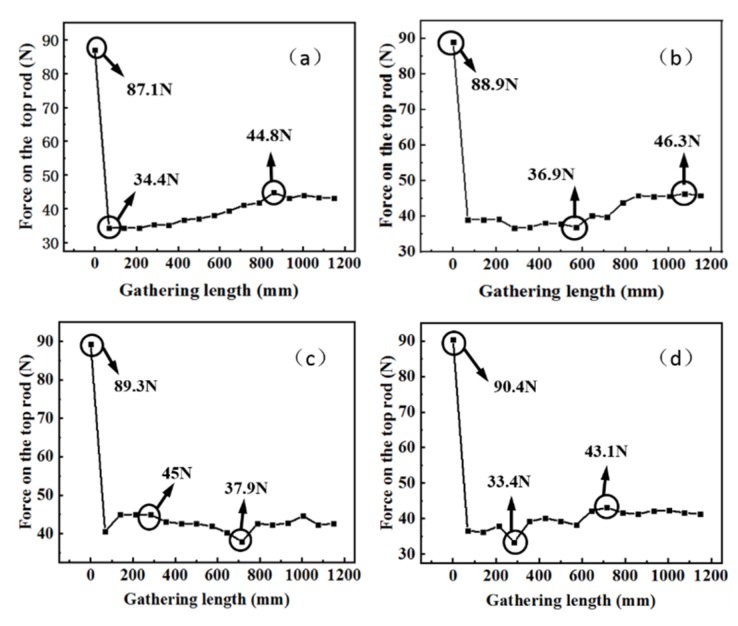
The force diagram of the top rod during the gathering process of the (**a**) first test; (**b**) second test; (**c**) third test; and (**d**) forth test.

In this test, the gathering process repeats four times with the force collection with a gathering speed of 0.042 m/s. The forces of the top rod are shown in Figure 15a–d. During all the four gathering processes, the initial force of the top rod is around 90 N, and the average force of the top rod during the gathering is about 40 N, except in the initial stage with an unstable state. The maximum force in four gathering processes is about 46.3 N and the minimum force is 33.4 N. The overall tendency of the force increases with the gathering length.

**Figure 16:**
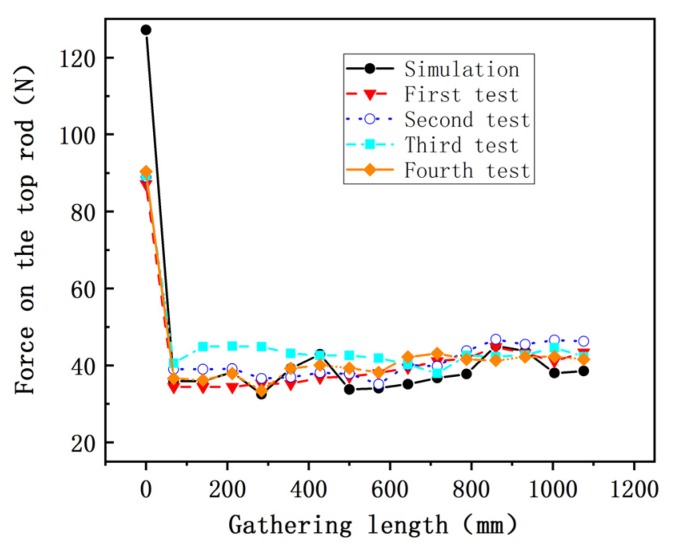
The comparison of finite element results and experimental data with the top rod load.

The experiment results demonstrate that STACER requires a constant force during gathering process with a gathering speed of 0.042 m/s. During the test, the friction force between layers of the spring reel changes under different test conditions. The force on the top rod fluctuates slightly which is less than 15% of the total force. It is similar to the force trend of the finite element result (from 32.4 to 45.1 N) with a top rod radius of 8.7 mm, as shown in Figure 16. The reason for this trend is that the surface friction force is increasing with the gathering length during the gathering process of a STACER. The consistency of the results from the experiment and finite element model verifies the reliability of the finite element model established in this work. This provides a guidance for the further mechanical simulation for the STACER.

## 6. Conclusions

An analytic model of the forming process of the STACER cylinder has been proposed to provide a general method for the design and production of a STACER from a theoretical level. A STACER finite element model has been established to simulate the mechanical characteristics and self-preservation performance during its gathering process by using finite element software. The effect of different radius dimensions of the top rod on the gathering process of the STACER has been studied. The influences of surface friction coefficient and acceleration direction on the gathered stability of the spring reel were analyzed. From this work, the following conclusions were drawn.

1. A different radius of the top rod affects the cone angle of the spring reel during gathering process. The large cone angle blocks the gathering process, and breaks the STACER. The optimum size of the top rod radius is 8.7 mm in this work. Local plastic deformation occurs at the riveting position when the radius of the top rod is 9.0 mm. When the radius of the top rod is 9.3 mm, the force on the top rod increases rapidly, which leads to the destruction of the spring reel after it is gathered.
2. The friction coefficient between the layers of spring reel is an important factor to effect the self-preservation of the STACER. When the friction coefficient is lower than 0.009, there is insufficient friction between the layers of the spring reel, and some layers move and deploy outwards of the storage barrel. When the friction coefficient is higher than 0.009, the spring reel can maintain a stable state.
3. Acceleration is another factor needs to be considered during the launching process of the STACER. When the acceleration direction is along the positive direction of X axis, the innermost layer near the top rod extends outward, and the gap between layers gradually increases. The friction decreases and some of the layers go beyond the innermost layer, and then exceed the top of storage barrel. Instead, only limited local areas appear in intermittent gaps when the acceleration is along other directions in the simulation. The STACER is in a stable state. Therefore, STACER should be installed without undertaking an acceleration along the positive X-axis direction at the riveting point during launching process.
4. The reliability and accuracy of the finite element model for simulating the mechanical characteristics and self-preservation performance of the STACER during the gathering process is verified by means of the experimental method. When the radius of the top rod is 8.7 mm, the tendency of the force on the top rod is similar between the simulation and test results. This provides a guidance for the further mechanical simulation of the STACER.
5. This study provides guidance for the design of all kinds of STACERs with different sizes and application conditions. For the diameter selection for the top rod, the radius of the top rod which equals the minimum end diameter of STACER is not suggested. It may cause serious deformation to the riveted part of STACER during the packaging process. For determining the friction coefficient, in the deployment process, a large friction coefficient helps STACER to achieve self-protection. With the consideration of gravitational acceleration, the directions affect the self-preservation of STACER and it is sensitive to a certain direction. So it is suggested that STACER is required to be installed in the proper direction for gravitational acceleration during its transportation or launching processes.

## Figures and Tables

**Table 1:** Specification of the STACER numerical model.

Name	Mesh Type	Number of Meshes	Parameters	Numerical Value
Top rod	R3D4	4000	Strip thickness	0.15 mm
STACER	S3R	88	Young’s modulus	2.05 × 10^5^ MPa
STACER	S4R	38842	Density	7.80 × 10^3^ kg/m^3^
Storage barrel	R3D4	9419	Poisson’s ratio	0.31
Spring reel	R3D4 + S3R + S4R	52349	Yield Strength	1.29 × 10^3^ MPa

## Supplementary Materials

The following are available online at https://www.mdpi.com/1996-1944/13/7/1650/s1, Figure S1: The spring reel with mesh sizes of (**a**) 0.99075 mm, (**b**) 2.06864 mm and (**c**) 3.03555 mm, Figure S2: The force on the top rod with different mesh sizes in the gathering processes.
