# Supplementary material for: A Study on the Mechanical Characteristics and Self-Preservation Performance of a Deployment Mechanism with a Large Exhibition Ratio during Its Gathering Process
Source: Materials (Basel). 2020 Apr 2;13(7):1650. doi: 10.3390/ma13071650 (PMC7178410; doi:10.3390/ma13071650)
Supplement: Supplementary file 1 [file materials-13-01650-s001.pdf]

materials

Supplementary Materials

A Study on Mechanical Characteristics and Self-Preservation Performance of the Deployment Mechanism with Large Exhibition Ratio during its Gathering Process

1. Mesh Sizes

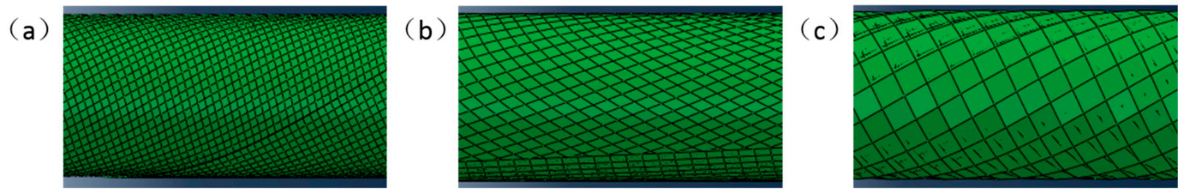

Figure S1. The spring reel with mesh sizes of (**a**) 0.99075 mm, (**b**) 2.06864 mm and (**c**) 3.03555 mm.

2. Mesh Sensitivity

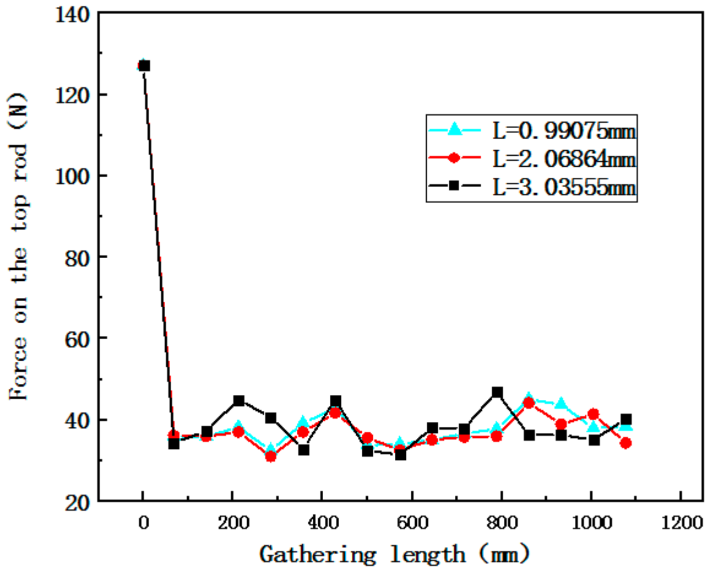

Figure S2. The force on the top rod with different mesh sizes in the gathering processes.
